# Moult Strategies Affect Age Differences in Autumn Migration Timing in East Mediterranean Migratory Passerines

**DOI:** 10.1371/journal.pone.0147471

**Published:** 2016-01-21

**Authors:** Yosef Kiat, Ido Izhaki

**Affiliations:** 1 Movement Ecology Laboratory, Department of Ecology, Evolution and Behavior, Alexander Silberman Institute of Life Sciences, Edmond J. Safra Campus, The Hebrew University of Jerusalem, Jerusalem, Israel; 2 Department of Evolutionary and Environmental Biology, University of Haifa, Haifa, Israel; Liverpool John Moores University, UNITED KINGDOM

## Abstract

Adult passerines renew their flight feathers at least once every year. This complete moult occurs either in the breeding areas, just after breeding (summer moult), or, in some long-distance migratory species, at the non-breeding areas, after arrival to the southern wintering area at the end of autumn migration (winter moult). The aim of this study was to relate moult strategies with the DMD, the difference in median migration date, through Israel, between juveniles and adults. Our data on autumn migration timing in juveniles and adults was based on ringing data of 49,125 individuals belonging to 23 passerine species that breed in Europe and Western Asia and migrate through Israel. We found that DMD was associated with moult timing. In all species that perform a winter moult, adults preceded juveniles during autumn. Among migrants who perform a summer moult, we found evidence of both migration timing patterns: juveniles preceding adults or adults preceding juveniles. In addition, in summer moulters, we found a significant, positive correlation between mean breeding latitude and DMD. Although previous studies described that moult duration and extent can be affected by migration, we suggest that moult strategies affect both migration timing and migration strategy. These two moult strategies (summer or winter moult) also represent two unique migration strategies. Our findings highlight the evolutionary interplay between moult and migration strategies.

## Introduction

The renewal of flight and body feathers is necessary to ensure future survival because old feathers, due to exposure to UV irradiation and other environmental factors, get worn, losing their functionality which thus affects fitness. All passerines moult all of their flight feathers at least once a year [1]. Moult is one of the three main energy-consuming events in the life cycle of migratory birds [2]. In most bird species, moult does not overlap with breeding and migration; however, in general, moult is more variable than breeding or migration in its timing within the annual cycle [2]. The most common moult strategy in passerines is moulting immediately after the breeding season. This strategy is employed by adults of all resident passerines in the Western Palearctic, as well as by all short-distance, migratory passerines wintering in the temperate zone, and also by some of the long-distance migrants wintering in equatorial or northern tropical regions [1]. Other long-distance migrants delay their moult until they reach their tropical winter areas [1–4].

Another moult strategy is the moult-migration strategy that is an offshoot of the summer moult strategy. In this strategy the migrants move from their breeding areas to another location, sometimes quite far from their breeding grounds, where they moult before resuming migration [5–7]. Because this moult strategy occurs among New-World passerines but not among Western Palearctic passerines, we do not address it in this study.

Seasons are not in alignment between the two hemispheres, such that the terms 'summer' or 'winter' are not absolute; in this paper the term 'summer moult' refers to complete post-breeding moult in the breeding area, while the term 'winter moult' refers to complete pre-breeding moult in the non-breeding, tropical winter areas, as per Jenni & Winkler [1].

Barta *et al*. [8] suggested that delaying of moult until arrival at the tropical winter areas occurs as a result of the short period of high food availability in the breeding grounds and a strong winter peak at other locations. Winter moults of long-distance migratory species were as long as summer moults of sedentary species and substantially longer than the rapid summer moult of long-distance migrants, which suggests that winter moult also allows Nearctic migrants to avoid the temporal constraints experienced during the post-breeding period [9]. The migration distance can influence the main moult period, summer or winter [9]. The evolution of the winter moult occurs together with the evolution of long-distance migration [10,11]. In addition, Hall & Tullberg [11] concluded that flexibility in the timing of moult might be a basal trait that could be a prerequisite for changes in migratory strategies.

Age dependent differences in the departure date of autumn migrants have been recorded in a wide range of bird species. In some long-distance migrants, the differences in departure dates between age groups is substantial [12]. Among passerines, the adult-juvenile difference in departure date seems to depend on whether a wing moult occurs in the breeding areas before migration onset ('summer moult'), or whether migration occurs immediately after breeding, with moult delayed until after arrival to the winter areas ('winter moult'). Based on these two moult strategies, one may expect that in species that moult before autumn migration, juveniles migrate first because they replace only their body feathers, a process which takes less time than that required for adults to replace their entire plumage. On the other hand, in species that postpone their moult to their non-breeding areas, it is expected that adults migrate first. Indeed, two studies in North America support this suggestion. Carlisle *et al*. [13] showed that in eight species that migrate immediately after breeding, before moulting, the adults migrate significantly earlier than juveniles, while for 27 species that perform a summer moult, the juveniles migrate earlier than adults. Benson & Winker [14] found that among seven passerines in Alaska that delayed the moult and migrated immediately after breeding, only in one species did the adults migrate earlier whereas in the six other species, no significant differences between adult and juvenile migration timing were found. In addition, in all 11 studied species that performed summer moult, the juveniles migrated before the adults [14]. Although Newton [12,15] suggested that similar patterns of moult strategies and migration timing also occur among European passerines, this has not yet been examined in detail.

Israel is one of the most important stopovers for birds migrating from their breeding sites in Europe and Western Asia to wintering grounds in Africa [16–18]. In this paper we used data collected on 49,125 individuals of 23 species, which included individual age and capture time in Israel, and for every species we added data on its breeding range and moult strategy to address the following questions: (1) Does the difference in the relative timing of migration between juveniles and adults vary between species with different timing of the adult's complete moult (summer or winter)? (2) For species that perform a summer moult before migration, does the difference in the timing of migration between juveniles and adults vary with migration distance between breeding areas and their stopover sites in Israel?

## Materials and Methods

**Fig 1:**
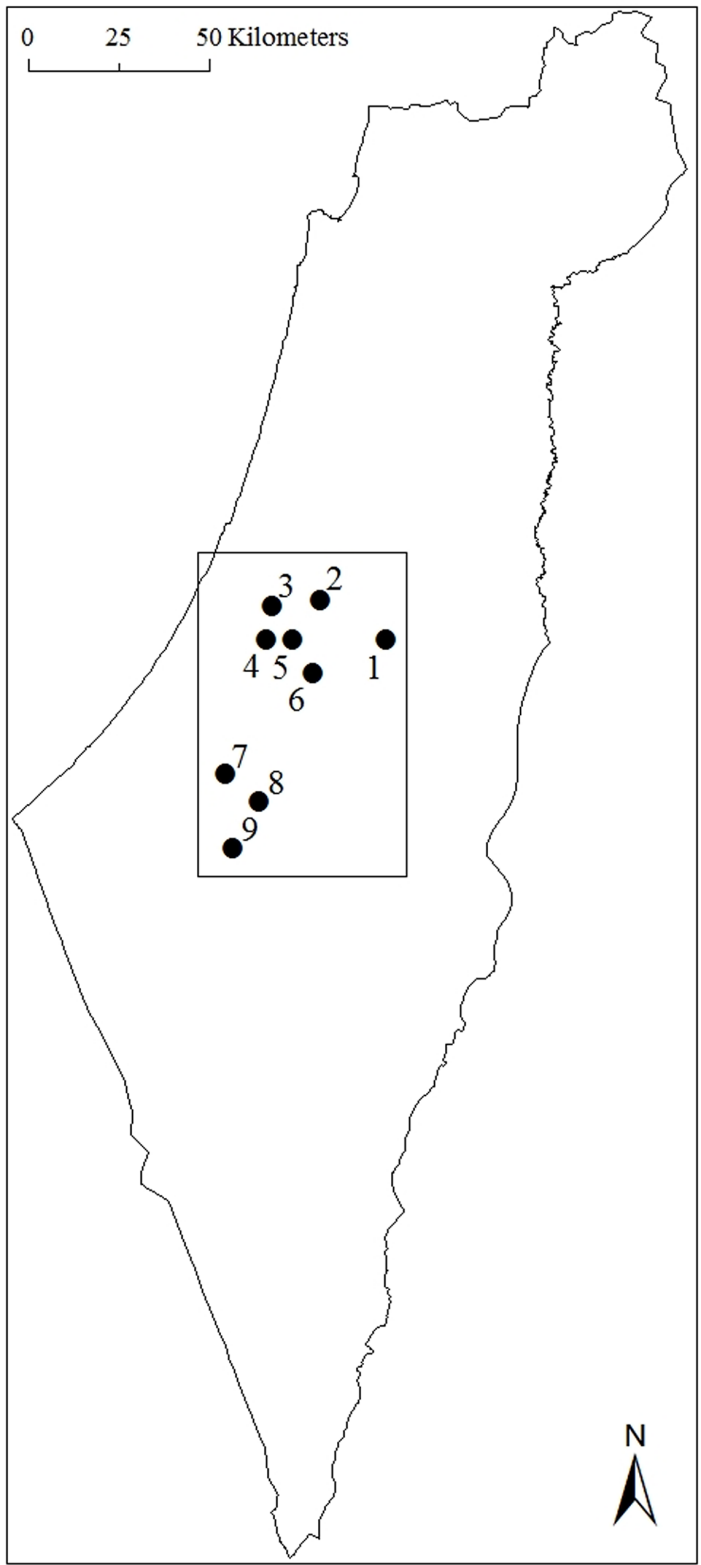
The ringing sites: (1) Jerusalem Bird Observatory 31°46'N / 35°12'E, (2) Modiin Hills 31°52'N / 34°59'E, (3) Einot-Gibton Nature Reserve 31°51'N / 34°52'E, (4) Tel-Ekron 31°46'N / 34°51'E, (5) Soreq Valley 31°46'N / 34°55'E, (6) Ella Valley 31°41'N / 34°58'E, (7) Beit-Qama Fields 31°26'N / 34°45'E, (8) Ein-Rimon 31°22'N / 34°50'E and (9) Be'er-Sheva Valley 31°15'N / 34°46'E.

Timing of autumn migration of adults and juveniles was calculated from the database of the Israeli Bird Ringing Center (IBRC) between 2008 and 2014. The data were collected during autumn migration, between July and November, at several ringing stations in central Israel, from the Modiin Hills (31°52'N / 34°59'E) in the north to the Be'er-Sheva Valley (31°15'N / 34°46'E) in south (Fig 1). The distance between the northernmost and southernmost sites (~70 km) is less than the migration distance of one day (Newton 2010). We calculated species-specific median migration dates for both adult and juvenile for 49,125 individuals of 23 species belonging to 15 genera. The median migration date was calculated on pooled data from all years and from all ringing stations with suitable data. All species included in this paper winter either in the Eastern Mediterranean region or in Africa. For each species, we calculated the difference in median migration date, through Israel, between juveniles and adults (DMD), as follows:

$$\text{DMD}=J_{median}-A_{median}$$ (1)

where J_*median*_ and A_*median*_ are the respective median dates that juveniles and adults pass through Israel during autumn migration.

We categorized each species either as a summer or winter moulter (S1 Table) based on published information for European passerines by Cramp [19], Svensson [20], Jenni & Winkler [1] and on our moult database collected in Israel. Species that perform a suspended or split moult or interrupt their post-breeding moult were categorized as summer or winter moulters by their main moult season. For example, although *Hirundo rustica* and *Riparia riparia* occasionally start their moult before the autumn migration they were considered here as winter moulters because this is their main moulting period [1]. *Sylvia hortensis* was considered as summer moulter because this species perform a split moult, in which most of the plumage is replaced before the autumn migration [21,22]. *Locustella luscinioides*, *Phylloscopus trochilus* and *Emberiza hortulana*, were also considered as summer moulters because they regularly perform a complete post-breeding moult at the breeding areas, with only a few individuals interrupting their moult and retaining a few old secondaries through the migration [1,23,24].

Although the definitions for long- or short-distance migrants in the literature is not clear [11], we established a classification method and each species was categorized as either a long- or short-distance migrant (S1 Table). We classified species as short-distance migrants if at least part of the population winters in the Eastern Mediterranean, whereas species whose wintering areas were entirely further south, in sub-Saharan Africa, were considered to be long-distance migrants. *Sylvia atricapilla* has an exceptional migration distribution as this species regularly winters in the Eastern Mediterranean but most of its population migrates through the Eastern Mediterranean and winters far in East Africa [22]. We considered this species to be a long-distance migrant. The mid latitude of the breeding area for each species was calculated from distribution maps published by Cramp [19].

### Statistical analysis

Differences in DMD between, species that moult in winter versus summer, were tested using a Mann-Whitney U-test. Species traits are known to be phylogenetically conserved [25]. To account for phylogenetic non-independence, we conducted all analyses using phylogenetic generalized least square (PGLS) regression [26] with moult strategy, mid breeding latitude and their interaction as the predictors (ANCOVA). We examined the strength of phylogenetic non-independence using the maximum likelihood value of the scaling parameter λ [27] implemented in the R package 'Caper' [28]. Pagel's λ is a multiplier of the off-diagonal elements of the variance-covariance matrix, which provides the best fit of the Brownian motion model to the tip data, and ranges between zero (no phylogenetic signal) and one (phylogenetic signal that depends on branch lengths as in analysis of phylogenetically-independent contrasts). The dependent variable for the PGLS analysis was the DMD. We then corrected for the effects on shared ancestry using the maximum value of λ. For a comprehensive review of comparative methods and their use in ecological and welfare studies see Mason [29]. We used AIC scores for model selection [30]. The phylogenetic tree was obtain using BirdTree.org [31]. All statistical analyses were done in R (v. 3.1.3; [32]).

## Results

### The relationship between DMD and moult strategy

**Fig 2:**
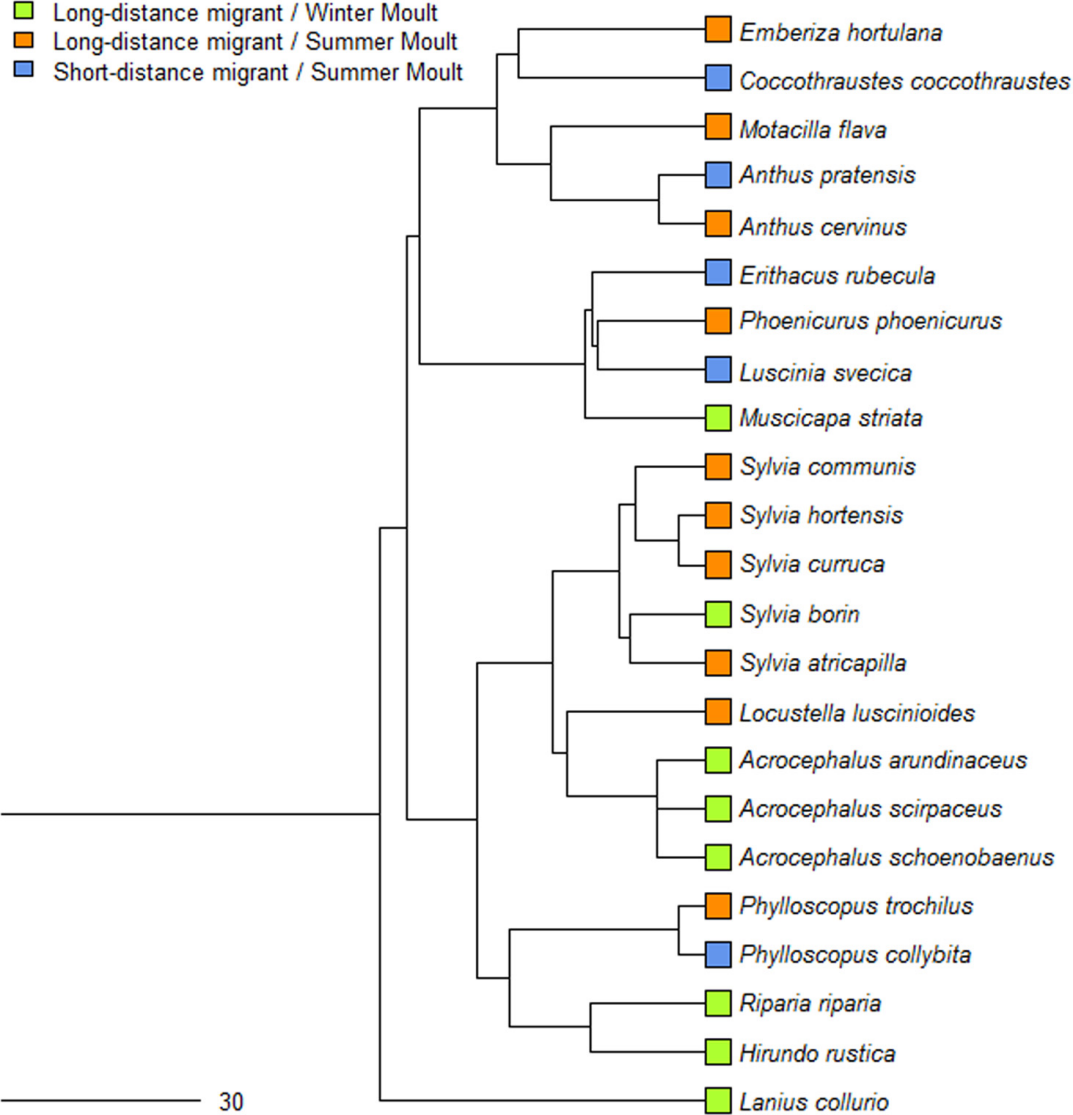
Phylogenetic tree for the 23 species included in analysis based on BirdTree.org [31].

**Fig 3:**
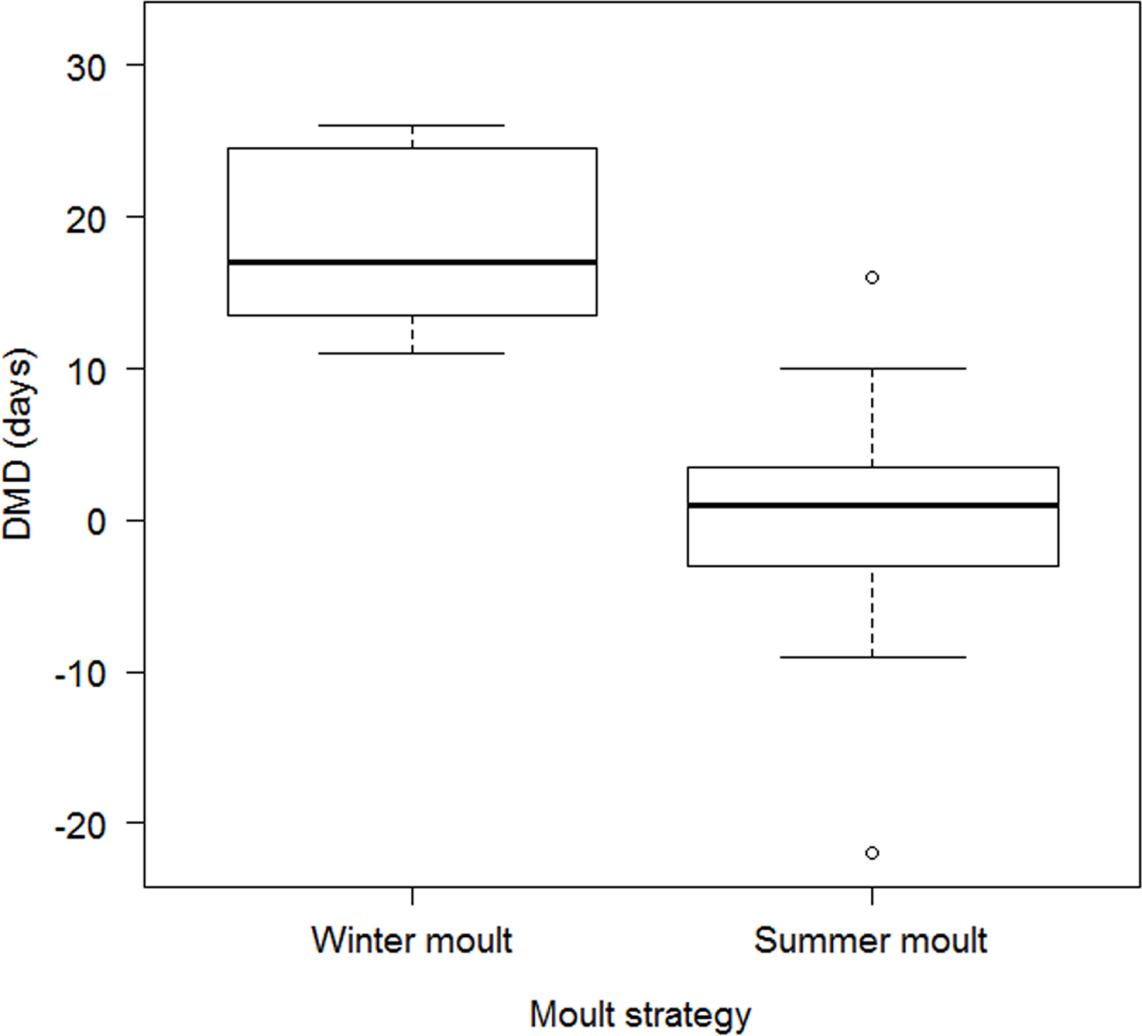
The difference in median migration date (DMD), through Israel, between juveniles and adults (days) in winter moulters (n = 8 species) and summer moulters (n = 15 species). The difference between the two groups was significant) Mann-Whitney U-Test, U = 3.00, Z = 3.69, P < 0.001).

We were able to extract data for calculation of the DMD value for 23 passerine species that breed in Europe or Western Asia and migrate through Israel (S1 Table), based on 49,125 individuals. The data indicated that species that perform a winter moult are all long-distance migrants (Fig 2). In all of these species, the adults preceded the juveniles during the autumn migration by 11–26 days, with a mean ± se DMD of 18.4 ± 2.1 days (n = 8 species; Fig 3). Species that perform a summer moult at the breeding area include both short-distance and long-distance migrants. For summer moulters, in five species, the juveniles preceded the adults and in 10 species the adults preceded the juveniles (S1 Table); the mean ± se DMD was 0.4 ± 2.3 days (n = 15 species; Fig 3). For more details, see the next section. The DMD for winter moulters was significantly larger than that of summer moulters (Mann-Whitney U-Test, U = 3.00, Z = 3.69, P < 0.001). The results from the PGLS regression indicated that there was only a low phylogenetic signal in the data (maximum likelihood value of λ = 0.08).

### The effects of moult strategy and breeding latitude on DMD

Our analysis showed that there were two models that best explain the differences in DMD values, as seen by their similar, low AICs (Mode 1: AIC = 152.07, F_3,19_ = 20.79, Adjusted R^2^ = 0.73, P < 0.001; Model 2: AIC = 152.08, F_2,20_ = 29.24, Adjusted R^2^ = 0.72, P < 0.001; Table 1). Model 1 included moult strategy, mid breeding latitude and their interaction as the predictors (Table 1). The DMD increased with latitude in the summer moult group (slope ± se = 1.07 ± 0.26, t_13_ = 4.13, P < 0.001), but not in the winter moult group (slope ± se = -0.15 ± 0.89, t_6_ = -0.17, P = 0.87). In species for which the median latitude of the breeding area is north of ca. 51°N, the adults preceded the juveniles (positive DMD values), but south of this latitude, the juveniles preceded the adults (negative DMD values). Such a correlation was not detected for species that perform winter moult (y = -0.15x + 25.85, r = 0.07, n = 8 species, P = 0.88). The intercept of the DMD of the summer moult group was -54.76 ± 13.45 days (95% confidence interval [-83.35, -26.17]), and that of the winter moult group was 25.85 ± 44.29 days (95% confidence interval [-86.21, 137.90]) but this difference was not statistically significant (t_19_ = 1.74, P = 0.10). The interaction between the predictors was not significant in either the summer or winter moult groups (t_19_ = -1.32, P = 0.204). The results from the PGLS regression indicated that there was no phylogenetic signal in the data (maximum likelihood value of λ = 0; Table 1).

**Table 1:** Statistical, phylogenetic signal (λ), AIC and ΔAIC for tested models.

Model	F_df_	R^2^	****P****	****λ****	****AIC****	****ΔAIC****
DMD ~ moult strategy * mid breeding latitude	20.79_3,19_	0.73	< 0.001	0.00	152.07	-
DMD ~ moult strategy + mid breeding latitude	29.24_2,20_	0.72	< 0.001	0.00	152.08	0.01
DMD ~ moult strategy	27.17_1,21_	0.54	< 0.001	0.08	162.81	10.73
DMD ~ mid breeding latitude	4.17_1,21_	0.13	0.054	1.00	175.60	12.79

**Fig 4:**
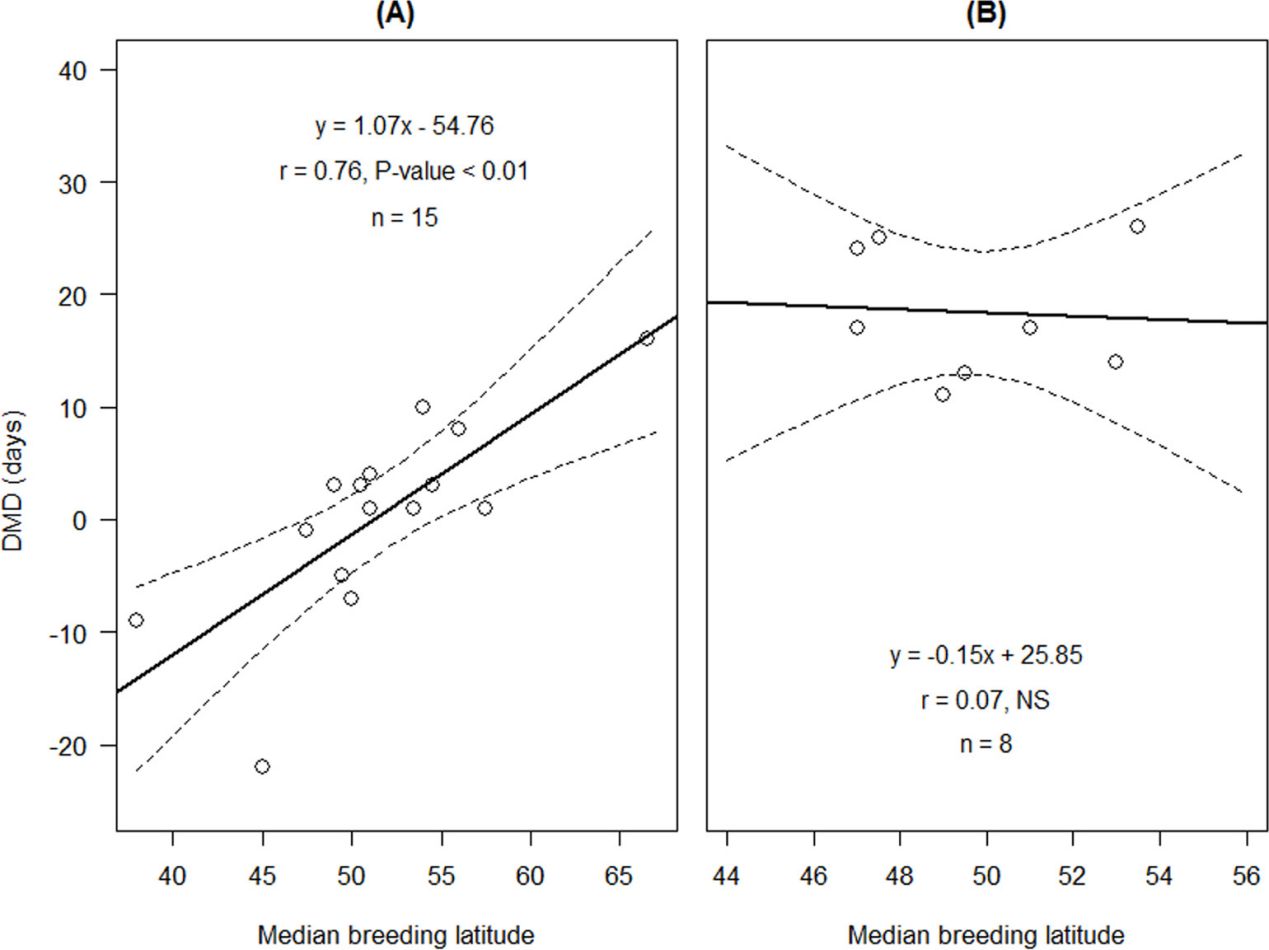
The difference in median migration date (DMD), through Israel, between juveniles and adults (days) in relation to breeding latitude and moult strategy, summer (A) or winter (B), in 23 passerines species. The difference between slopes is significant (P < 0.05); dashed curves represent 95% regression confidence intervals.

Our results among migrants who perform a summer moult indicated a positive correlation between breeding latitude and DMD (y = 1.07x − 54.76, r = 0.76, n = 15 species, P < 0.01). Thus, the differences increased with breeding latitude (Fig 4).

### Ethical approval

All applicable international, national, and/or institutional guidelines for the care and use of animals were followed.

## Discussion

### Winter moult and migration strategy

Delaying the complete post-breeding moult until arrival at the wintering areas occurs only among long-distance migrants [1]. This moult strategy probably evolved from the basic strategy of post-breeding summer moult before autumn migration [10,33]. Thus, winter moult may be considered as an adaptation to long-distance migration. For species that defend winter territories, there is an advantage to delaying the post-breeding complete moult until arrival at the winter areas as these species are under selective pressure to complete a rapid migration and arrive quickly to their wintering site [34,35]. Carlisle *et al*. [13] and Newton [12] noted that the time required for adult birds to moult and the location of the moult contribute heavily to the age differences in migration departure date. Our results indicate that among Western Palearctic passerines, winter moult is found only in long-distance migrants (Fig 2) and is associated with adults preceding juveniles in migration (positive DMD values; Fig 3). In all of the species which perform a winter moult, adults preceded the juveniles during the autumn migration by more than two weeks. Similar results have been reported for migratory passerines in western North America [13]. No relation was found between breeding latitude and DMD among long-distance migrants that perform winter moult (Fig 4). Although there is an advantage to reaching wintering areas earlier in order to claim and defend territories, the adults of those, which perform winter moult do not need to reduce the time difference between their migration and juvenile migration because the postponement of moulting until the winter already produces this timing difference, and thus adults reach wintering areas first.

### Summer moult and migration strategies

Both migration patterns, adults preceding juveniles and juveniles preceding adults, occur in species who perform a summer moult (positive and negative DMD values, respectively, Fig 3). For these species, we found a positive relationship between median breeding latitude and DMD. Thus, in species that breed in more northern regions (ca. > 51°N), adults preceded juveniles whereas in species breeding in more southern regions (ca. < 51°N) juveniles preceded adults (Fig 4). This result can be explained by evidence of higher migration efficiency of adults in comparison with juvenile passerines. Ellegren [36] showed that for *Luscinia svecica* in eastern Sweden, adult birds migration requires less time than juveniles migration. Carlisle *et al*. also found evidence that New World, summer-moulting adults migrated faster than juveniles [13]. This higher speed could be the result of higher efficiency in foraging and fattening in stopover sites of adults, which leads to a shorter stopover duration for adults than for juveniles [36–39]. Furthermore, the summer moult, before migration, provides fresh flight feathers which increase flight efficiency [7,40].

Therefore, we speculate that in summer moulters, the migration departure of adults occurs later as they perform a complete moult. In species that migrate to Israel from nearby countries (southern latitudes), the juveniles arrive earlier because they depart earlier and have a "head start". In species from northern countries the adults have the opportunity to overtake the juveniles by the time they reach central Israel. This hypothesis can explain the difference we showed in DMD values. Our sample represented species from a large geographic range, and their (sub)populations were not separated in this study. In addition, in our sample, there were six species for which a portion of their population winters in Israel; however, the wintering populations are much smaller than the migratory populations [18]. Furthermore, in this study, we only included individuals that were mist-netted during their autumn migration period (July-November) [18]. Unintentional inclusion of wintering individuals, through limited, could have added some noise to the results, thus making the application our results more conservative.

We also suggest that summer and winter moult represent two unique migration strategies. In birds that delay the moult to the wintering areas, the adults preceded the juveniles; they start the migration before the juveniles and stay ahead of them until arrival to the wintering areas. The alternative strategy is a summer moult and migration starting at the same time or later than juveniles. This strategy requires the extra cost of a higher speed journey for the adults in order to arrive at the wintering areas before the juveniles which is thus associated with higher energetic costs. The intensity of winter territoriality may affect migration speed considerations. In this study, we did not consider the intensity of territoriality due to the difficulty of obtaining the data from wintering areas of most species and of quantifying territoriality, but this may be interesting to study in the future.

We only found a slight phylogenetic signal in our focal species. This slight signal in relation to moult strategies, summer or winter, is expected because of the high mixing of these two strategies in some passerine families (e.g. *Hirundinidae*, *Sylviidae*, *Muscicapidae* and *Laniidae*). For example, through phylogenetic analysis, Svensson & Hedenstrom [10] found that winter moult evolved 7–10 times just within the Sylviidae family.

Although previous studies found that moult duration and extent can be affected by migration [41,42], our results added to prior findings [7,13] that imply that the timing of the main moult (summer or winter) can influence both migration timing and migration strategy. This highlights the evolutionary interplay between moult and migration strategies.

## Supporting Information

S1 Table Median migration dates through stopover sites in central Israel (East Mediterranean) for adult and juvenile passerines with different moult and migration strategies. (PDF)
