# Supplementary material for: Moult Strategies Affect Age Differences in Autumn Migration Timing in East Mediterranean Migratory Passerines
Source: PLoS One. 2016 Jan 21;11(1):e0147471. doi: 10.1371/journal.pone.0147471 (PMC4721648; doi:10.1371/journal.pone.0147471)
Supplement: S1 Table — (PDF) [file pone.0147471.s001.pdf]

	Moult strategy of adults (S-summer, W-winter)	Migration distance (S-short, L-long)	Adults median migration date	Juvenile median migration date	Number of days between adults and juvenile median migration	Sample size	
						Adults	Juveniles
*Riparia riparia**	W	L	09-Sep	26-Sep	17	252	616
*Hirundo rustica**	W	L	23-Sep	04-Oct	11	1243	4965
Anthus pratensis	S	S	13-Nov	21-Nov	8	145	366
Anthus cervinus	S	L	14-Oct	30-Oct	16	464	425
Motacilla flava	S	L	14-Sep	08-Sep	-6	1153	4347
Erithacus rubecula	S	S	07-Nov	10-Nov	3	264	813
Luscinia svecica	S	S	26-Oct	29-Oct	3	497	1125
Phoenicurus phoenicurus	S	L	12-Oct	16-Oct	4	615	955
*Locustella luscinioides***	S	L	27-Sep	05-Sep	-22	81	289
Acrocephalus schoenobaenus	W	L	02-Sep	28-Sep	26	169	217
Acrocephalus scirpaceus	W	L	27-Aug	20-Sep	24	611	1972
Acrocephalus arundinaceus	W	L	02-Sep	27-Sep	25	230	330
Sylvia borin	W	L	08-Sep	22-Sep	14	74	217
Sylvia atricapilla	S	L	11-Sep	21-Sep	10	1374	3732
*Sylvia hortensis****	S	L	21-Aug	12-Aug	-9	424	1544
Sylvia curruca	S	L	02-Sep	28-Aug	-5	1537	4121
Sylvia communis	S	L	20-Sep	13-Sep	-7	76	219
*Phylloscopus trochilus***	S	L	25-Sep	26-Sep	1	2054	6418
Phylloscopus collybita	S	S	06-Nov	07-Nov	1	1078	2656
Muscicapa striata	W	L	11-Sep	28-Sep	17	93	327
Lanius collurio	W	L	13-Sep	26-Sep	13	114	529
Coccothraustes coccothraustes	S	S	10-Nov	13-Nov	3	64	67
*Emberiza hortulana***	S	L	12-Sep	13-Sep	1	105	158

* Part of population perform suspended moult, most plumage renew in south.

** Summer complete or almost complete.

*** Split moult, most plumage renew in north.
